# Molecular prevalence of trypanosome infections in cattle and tsetse flies in the Maasai Steppe, northern Tanzania

**DOI:** 10.1186/s13071-017-2411-2

**Published:** 2017-10-23

**Authors:** Mary Simwango, Anibariki Ngonyoka, Happiness J. Nnko, Linda P. Salekwa, Moses Ole-Neselle, Sharadhuli I. Kimera, Paul S. Gwakisa

**Affiliations:** 1 Department of Veterinary Medicine and Public Health, College of Veterinary Medicine and Biomedical Sciences, Sokoine University of Agriculture (SUA), P.O box 3015, Morogoro, Tanzania; 2 Nelson Mandela African Institution of Science and Technology, School of Life Sciences and Bioengineering, P. O. Box 447, Arusha, Tanzania; 3 Department of Geography and Environmental Studies, University of Dodoma, P. O. Box 395, Dodoma, Tanzania; 4 Genome Sciences Centre, Department of Microbiology, Parasitology and Biotechnology, College of Veterinary Medicine and Biomedical Sciences, Sokoine University of Agriculture, Morogoro, Tanzania; 5 FAO Emergency Centre for Transboundary Animal Disease (ECTAD), P.O Box 2, Dar es Salaam, Tanzania

**Keywords:** Trypanosomes, Molecular prevalence, Cattle, Tsetse flies, Maasai Steppe, Tanzania

## Abstract

**Background:**

African trypanosomosis is a disease of public health and economic importance that poses a major threat to the livelihoods of people living in the Maasai Steppe, where there is a significant interaction between people, livestock and wildlife. The vulnerability of the Maasai people to the disease is enhanced by the interaction of their cattle, which act as vehicles for trypanosomes, and tsetse flies close to wildlife in protected areas. This study was aimed at identification of trypanosome infections circulating in cattle and tsetse flies in order to understand their distribution and prevalence in livestock/wildlife interface areas in the Maasai Steppe.

**Methods:**

A total of 1002 cattle and 886 tsetse flies were sampled from June 2015 to February 2016 in five villages and PCR was conducted to amplify the internal transcribed spacer 1 (ITS1) from trypanosomes. All *Trypanosoma brucei*-positive samples were further tested for the presence of the serum resistance-associated (SRA) gene found in human-infective trypanosomes using the SRA-LAMP technique.

**Results:**

The overall prevalence of trypanosome infections was 17.2% in cattle and 3.4% in tsetse flies. Using a nested PCR, prevalence and abundance of five trypanosome species, *Trypanosoma vivax*, *T. brucei*, *T. simiae*, *T. theileri* and *T. congolense*, were determined, which varied with season and location. The highest prevalence of the identified trypanosome species was recorded at the end of wet season with an exception of *T. brucei* which was high at the beginning of the wet season. No human-infective trypanosomes were detected in both cattle and tsetse fly DNA.

**Conclusions:**

This study confirms that seasonality and location have a significant contribution to the prevalence of trypanosome species in both mammalian and vector hosts. These results are important for designing of community-wide vector and disease control interventions and planning of sustainable regimes for reduction of the burden of trypanosomosis in endemic pastoral areas, such as the Maasai Steppe in northern Tanzania.

## Background

African trypanosomosis is a disease of public health importance that affects humans and animals. Animal African trypanosomosis (AAT) causes reduced production and animal losses leading to reduced economic growth [1, 2] of the Maasai people of northern Tanzania. The Maasai pastoralists depend mostly on livestock production for their livelihood, but trypanosomosis is a significant problem in the interface areas due to proximity to wildlife reservoirs. The distribution of African trypanosomosis corresponds to the distribution of tsetse flies transmitting the disease [2, 3]. In Tanzania, *Glossina* spp*.* are widely distributed across the country especially in protected areas, game reserves, national parks and wildlife corridors such as the Maasai Steppe. Tsetse density has been shown to be high in the Manyara, Mara and Tanga regions of Tanzania, and *Glossina morsitans* is the most widely distributed tsetse fly species [4]. Subspecies of *G. morsitans* and *G. fuscipes* have been reported in western Tanzania while *G. pallidipes* and *G. swynnertoni* were reported to be most abundant in northern Tanzania [4, 5]. These species transmit trypanosomes of importance in Africa including *T. congolense*, *T. suis*, *T. vivax*, *T. simiae*, *T. equipedum*, *T. evansi*, *T. godfreyi* and *T. brucei* [3, 6, 7]. These species are widely distributed in animal and human hosts. They cause disease in a wide range of domestic animals including cattle, pigs, camels, goats, sheep, dogs, cats, horses and monkeys [6, 8, 9].

In East Africa, trypanosomosis has been endemic for a long time following a history of epidemics that occurred in the past [10]. In northern Tanzania, the long standing endemicity of the disease is due to ecological factors and vector biology that support the persistent circulation of trypanosomes [6, 11]. The ability of tsetse flies to transmit trypanosomes is facilitated by the environment in which the vectors interact with wildlife reservoirs and vulnerable populations in wildlife/livestock/human interface areas. Such an environment provides a suitable setting for transmission and spread of African trypanosomes [12]. The transmission of trypanosomes to susceptible animals and humans is further enhanced by climatic conditions and human activities associated with livestock movement and control regimes in the Maasai Steppe ecosystem [13–17]. Such practices maintain trypanosome circulation in vectors and livestock by creating carriers, which may not be detected in a population due to lack of clinical symptoms. However, molecular diagnostic techniques have made it possible to detect and identify infections even prior to the manifestation of the disease [6, 18]. The polymerase chain reaction has successfully been used for detection and characterization of infectious agents [19]. Several oligonucleotide primers have been developed to amplify trypanosome genes, some of which are generic or species-specific. The sensitivity of these primers has been reported previously [20–23].

Due to insufficient knowledge on the current status of trypanosomosis, there are no sustainable means or uniform vector control regimes suitable for interface areas, and this situation increases the vulnerability of humans and their livestock to trypanosomosis. Therefore, understanding of current infection status is key to providing the required knowledge for controlling African trypanosomoses appropriately. The aim of this study was to quantify trypanosome infections circulating in cattle and tsetse flies and understand their distribution and prevalence in livestock/wildlife interface areas of the Maasai Steppe, Tanzania.

## Methods

### Study area

The study was conducted in the Maasai Steppe of northern Tanzania, made up of Simanjiro plains, Tarangire National park and Lake Manyara National park [24]. It lies between 3°52′ and 4°24′S and 36°05′ and 36°39′E. The area has two rainfall seasons of spatial and temporal variation, comprising of short rains between October and December and long rains from February to May. The average temperature in the area is between 18 °C and 30 °C. The Maasai Steppe is made up of natural semi-arid ecosystem providing a home for a variety of animal species, vegetation, conducive temperature variations and rainfall, all of which support a natural habitat for vectors and various parasitic organisms [25]. Livestock and crop production are the main sources of livelihood in the Maasai Steppe with cattle production being the major activity [26]. Due to its proximity to the protected areas, the study area has the high interaction of wild, domestic animals and humans which increases circulation of trypanosomes through bites from tsetse flies, making trypanosomosis endemic in the area.

### Study design

**Fig. 1:**
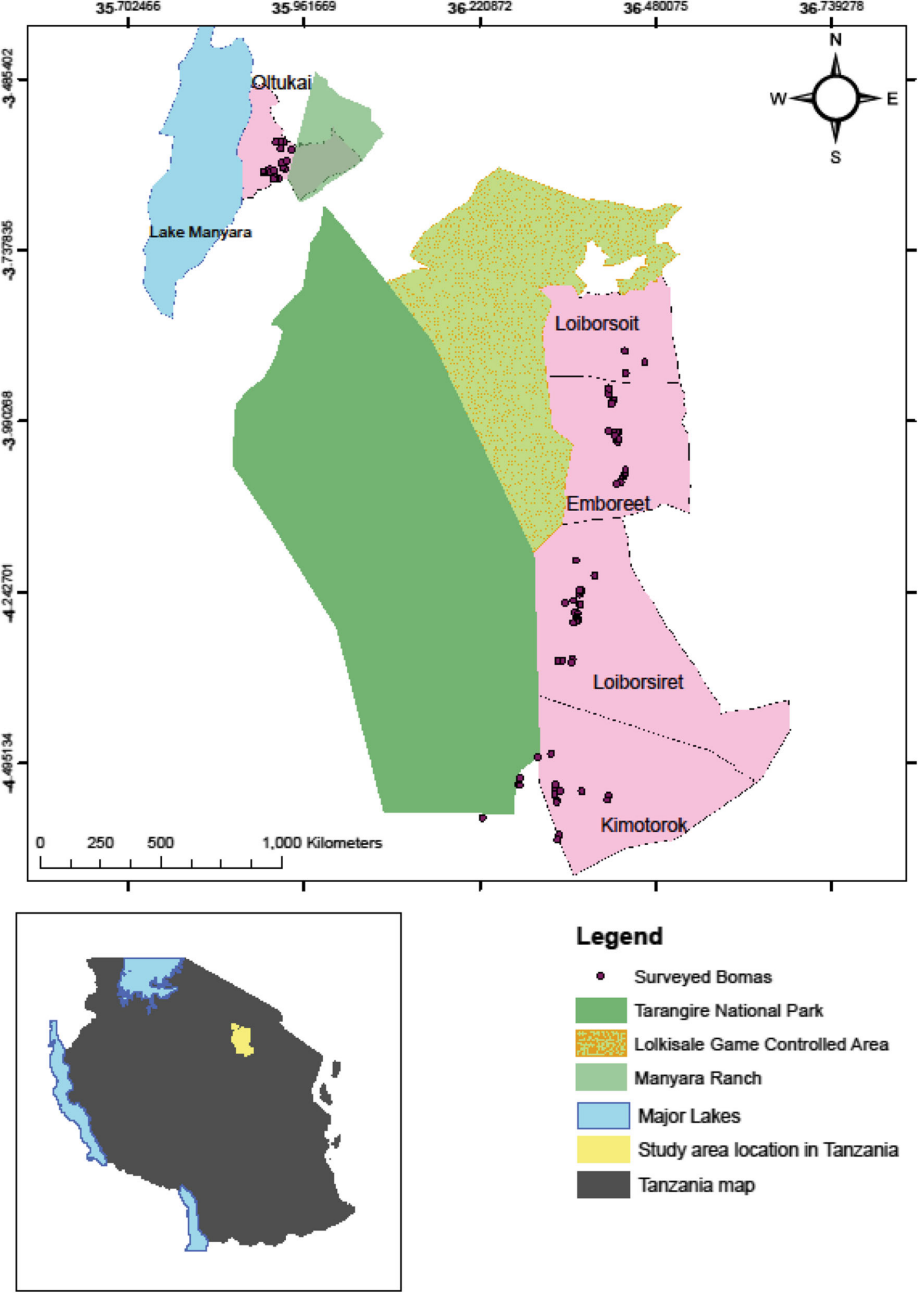
Map showing the study villages in Simanjiro and Monduli districts of northern Tanzania

A repeated cross-sectional, observational study design was used in which three samplings were conducted. A multistage cluster sampling technique was used to select cattle whereby Lake Manyara and Tarangire National parks were used as land marks for the study. The sampling allowed a comparison of the magnitude of trypanosome infections between the end of wet (June 2015), dry (August 2015) and the beginning of wet (February 2016) seasons. Blood samples were collected from 1002 cattle in five villages (Fig. 1): Emboreet (*n* = 340), Loiborsiret (*n* = 200), Kimotorok (*n* = 200), Loiborsoit (*n* = 60) and Ortukai (*n* = 202). The five villages were selected based on their proximity to Tarangire National park, in the same areas where tsetse flies were captured. In each village, 50% of sub-villages were selected at random, and 50% of all bomas in each sub-village were selected systematically. A boma was taken as a sampling unit in which animals were selected at random. A boma is a traditional Maasai homestead, usually consisting of a number of huts surrounding an enclosure for livestock, especially cattle. The average number of cattle sampled per boma was seven, and a total of 94 bomas were selected. Sample size calculation was based on the assumption of a 95% confidence level, 27.5% [11] expected trypanosome parasite prevalence, 0.05 tolerable error and a design effect of 1.2 for cluster sampling.

### Blood sample collection and trapping of tsetse flies

Blood samples were taken from each animal by jugular venipuncture using 10 ml vacutainer tubes (Becton Dickinson Vacutainer Systems, Plymouth, UK) containing ethylenediaminetetraaceticacid (EDTA) anticoagulant. Blood samples were then kept in cool boxes on ice for 2–5 h before refrigeration. The blood samples were further stored in a freezer at -20 °C until the day of DNA extraction.

A total of 886 tsetse flies were collected in June 2015 using Epsilon traps odour-baited with an attractant made from acetone, phenols and octanol. Traps were set at 27 sites in the study villages for a month, where they were checked and emptied every six days. Each trapped fly was kept in a separate Eppendorf tube with 70% ethanol at room temperature until DNA extraction.

### DNA extraction

DNA was extracted from cattle blood as described in the protocol by Quick-gDNA blood mini prep kit (Zymo Research, Irvine, CA, USA). Ground tsetse flies were individually put in a ZR Bashing Bead™ Lysis Tube, and DNA was extracted using the protocol by ZR Tissue and Insect DNA Mini Prep, (Zymo Research). Fifty microlitres of DNA was eluted in Eppendorf tubes and stored at -20 °C until further analysis.

### PCR identification of trypanosome species

A nested PCR was employed using two sets of primers comprising of outer forward (5′-GAT TAC GTC CCT GCC ATT TG-3′) and reverse (5′-TTG TTC GCT ATC GGT CTT CC-3′) primers, and inner forward (5′-GGA AGC AAA AGT CGT AAC AAG G-3′) and reverse (5′-TGT TTT CTT TTC CTC CGC TG-3′) primers [20, 22]. The nested PCR (nPCR) reaction was conducted in a 12.5 μl reaction comprising of 6.25 μl mastermix (Quick-Load *Taq* 2× Master Mix, New England BioLabs Inc., Ipswich, MA, USA), 2.5 μl DNA template, 3.25 μl nuclease free water and 0.2 μM of each first round primer. The second round amplification was performed using primers at the same concentration as the first round, 1 μl of the PCR products from the first round was used as a template and 4.75 μl of nuclease free water. Amplification conditions involved an initial denaturation step at 95 °C for 7 min followed by 35 cycles of denaturation at 94 °C for one min, an annealing step at 55 °C for one min, then extension step of 72 °C for two min and a final extension at 72 °C for 10 min. The PCR products were loaded on 1.5% agarose gel stained with GRGreen Nucleic Acid Stain (Excellgen, Inc., Rockville, MA, USA) and visualized on a Gel DocTM (Bio Rad, Hercules, CA, USA).

Additionally, a single step PCR for the identification of ITS 1 gene was used to compare the prevalence of trypanosome infections in cattle and tsetse flies. Briefly, primers with the following sequences were used: F: 5′-CCG GAA GTT CAC CGA TAT TG-3′ and R: 5′-TTG CTG CGT TCT TCA ACG AA-3′ [27]. The reaction was performed in a 25 μl reaction volume containing 12.5 μl of mastermix (Quick-Load *Taq* 2× Master Mix, New England BioLabs Inc., Ipswich, MA, USA) containing DreamTaq DNA polymerase supplied in 2× DreamTaq buffer, 0.4 mM of each of the dATP, dCTP, dGTP and dTTP, and 4 mM MgCl_2_, 0.2 μM of each of the forward and reverse primers, 6.3 μl nuclease free water and 5 μl DNA template. The reaction was conducted in a thermocycler (ProFlex PCR system, Applied Biosystems, Foster City, CA, USA) with an initial denaturation step of 94 °C for 3 min, followed by 30 cycles of 94 °C for 30 s, 55 °C for 30 s, 72 °C for 30 s and a final extension step at 72 °C for 10 min [28]. Expected fragment size for individual trypanosome species is shown in Table 1.

**Table 1:** Expected band sizes of trypanosome species amplified by two methods

Trypanosome species	ITS1 PCR (bp)	Nested ITS PCR (bp)
*T. congolense* forest	710	1513
*T. congolense* savannah	700	1413
*T. congolense kilifi*	620	1422
*T. brucei*	480	1207–1224
*T. simiae*	400	850
*T. vivax*	250	611
*T. theileri*		988

### SRA-LAMP for detection of human-infective trypanosomes

All *T. brucei* positive DNA from cattle and tsetse flies were subjected to SRA-LAMP technique to detect human-infective trypanosomes (*T. b. rhodesiense*) [29]. The technique was performed using the primer sequences shown in Table 2. SRA-LAMP was conducted in 25 μl reaction volume containing 2.5 μl of 1× Thermopol buffer, 5 μl of 0.8 M betaine, 1 μl of 200 μM dNTPs, 1 μl of 0.2 μM F3 and B3 primers, 1 μl of 2 μM FIP and BIP primers, 1 μl of 0.8 μM loop primers LB and LF, 1 μl of 8 U Bst DNA polymerase, 2 μl of DNA template and 7.5 μl of nuclease free water. Reaction conditions for the SRA-LAMP were set at 62 °C for 1 h in a GeneAmp® PCR system 9700 (Applied Biosystems, Foster City, CA, USA). The reaction was terminated by increasing the temperature to 80 °C for 5 min. The SRA-LAMP products were visualised through colour change after addition of 2 μl SYBR Green I. An orange colour indicated negative results while the colour change to green signified positive results. The SRA-LAMP products were confirmed by gel electrophoresis using 1.5% agarose gel prepared in 1× TBE and stained with 1 μl of 10 mg/ml ethidium bromide. The products were separated at 120 V for 30 min and visualised using Ultra violet trans-illuminator (UVDoc™ Merton, UK).

**Table 2:** SRA-LAMP primer sequences

Primer	Primer type	Primer sequence (5′–3′)
SRA-F3	F3	GCGGAAGCAAGAATGACC
SRA-B3	B3	TCTTACCTTGTGACGCCTG
SRA-FIP	FIP	GGACTGCGTTGAGTACGCATCCGCAAGCACAGACCACAGC
SRA-BIP	BIP	CGCTCTTACAAGTCTTGCGCCCTTCTGAGATGTGCCCACTG
SRA-LF	LF	CGCGGCATAAAGCGCTGAG
SRA-LB	LB	GCAGCGACCAACGGAGCC

### Data analysis

The prevalence of infections was estimated using frequency and contingency tables in Excel and Epi Info™ version 7.0 (CDC, Atlanta, GA, USA) statistical software. Comparative location and seasonal prevalence were estimated using contingency tables, and logistic regression where the Chi-square expected and *P*-values were obtained. The chi-square values were obtained by a test of independence and homogeneity of proportions between dependent (outcome) and independent (exposure) variables using Pearson uncorrected test. Chi-square was used when all expected values were greater than five while Mid-*P* values for two-way tables were obtained when at least one expected value (row total*column total/grand total) was less than five. All *P*-values for tables with zero values were obtained with the assumption that 0.5 was added. The *P*-values of the results were analysed (using *P* = 0.05 as a cut-off value) at 95% confidence intervals to indicate the level of uncertainty around the obtained values.

## Results

### Prevalence of trypanosome infections in cattle

**Fig. 2:**
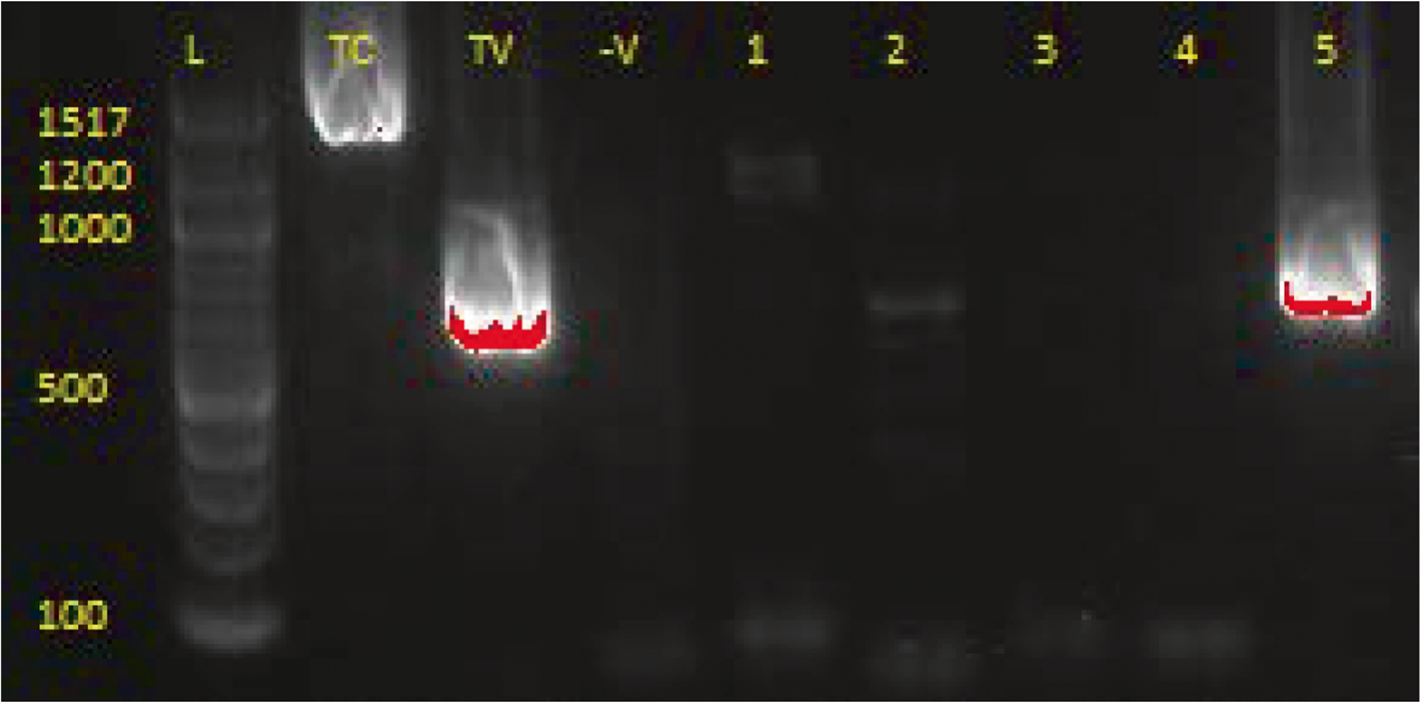
A gel picture of trypanosomes amplified using nPCR. Lane L: a 100 base pair (bp) DNA ladder; Lanes TC and TV: positive controls for *T. congolense* and *T. vivax*, respectively; Lane -V: negative control; Lanes 1–5: cattle DNA samples. Lane 1 shows a weak *T. brucei-*positive sample, Lanes 2 and 5 are *T. vivax-*positive samples and Lanes 3 and 4 are negative samples

The overall prevalence of trypanosome infections in cattle detected using the nested PCR assay (Fig. 2) was 17.2% (172/1002; 95% CI: 14.91–19.68) (Table 3).

**Table 3:** Prevalence of trypanosome infections in the study population of cattle by village, age and sex

Category	Sub-category	No. of cattle screened	No. of trypanosome infections	Prevalence (%)	*χ* ^*2*^	*P*-value
Village	Emboreet	340	41	12.1	21.42	0.0003
	Kimotorok	200	41	20.5		
	Loiborsiret	200	36	18.0		
	Loibor-soit-A	60	21	35.0		
	Ortukai	202	33	16.3		
	Total	1002	172	17.2		
Age (years)	< 2	301	51	16.9	32.91	0.0631
	2–6	484	79	16.3		
	6–10	212	40	18.9		
	> 10	5	2	40.0		
	Total	1002	172	17.2		
Sex	Female	716	125	17.5	0.15	0.6977
	Male	286	47	16.4		
	Total	1002	172	17.2		

Prevalence of trypanosome infections varied between the five different villages significantly (*P* = 0.0003). Thus, the highest prevalence was found in Loibor-soit-A village (35.0%) followed by Kimotorok (20.5%), whereas lowest prevalence (12.1%) was observed in Emboreet village. The differences in prevalence between Kimotorok and Emboreet (*P* = 0.009) as well as Loibor-soit-A and Emboreet (*P* < 0.0001) were significant. No statistical significance was however observed when the prevalence of trypanosome infections was compared between different age or sex groups, although a strong association between prevalence and age of the animal was documented (*χ* ^2^ **=** 32.91, *P* = 0.0631). Thus, highest prevalence (40.0%) was observed in cattle aged older than 10 years, whereas lowest prevalence (16.3%) was detected in cattle aged between 2 and 6 years and those younger than 2 years. Although infection prevalence was higher in female cattle (17.5%) than in males (16.4%), this difference was not statistically significant (Table 3).

### Abundance of single and mixed trypanosome infections in cattle

**Fig. 3:**
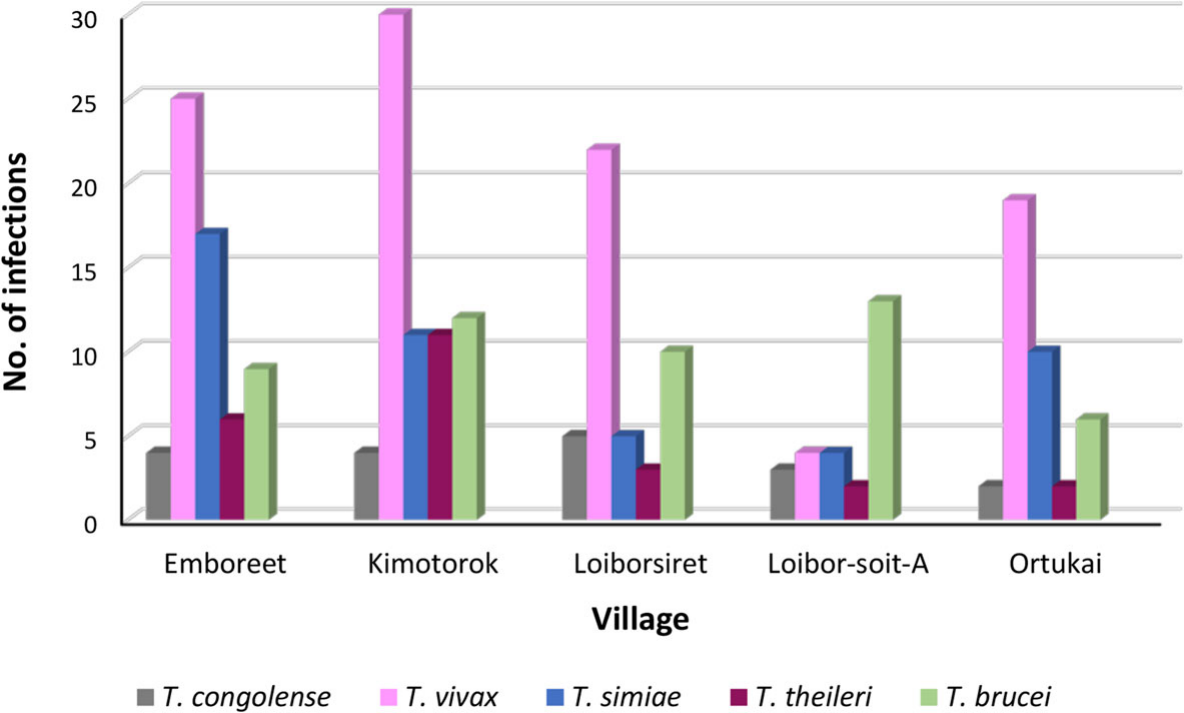
Spatial distribution of trypanosome species in the Masaai Steppe

A total of 239 trypanosome infections were scored in the 172 positive cattle. While most cattle were infected with single trypanosome species (115/172 cattle; 66.9%), 27.9% (48/172) of the trypanosome-infected cattle carried multiple infections with two trypanosome species, 4.7% (8/172) carried three trypanosomes, and one animal (1/172; 0.6%) was infected with four trypanosome species. The most frequent multiple infections were *T. vivax*/*T*. *simia*e occurring in 12.8% (22/172) of infected cattle followed by *T. brucei*/*T. vivax* (5.2%; 9/172). *Trypanosoma vivax* was the most abundant species (100/239), followed by *T. brucei* (50/239), *T. simiae* (47/239), *T. theileri* (24/239) and *T. congolense* (18/239). *Trypanosoma vivax* was the most abundant in all villages except in Loibor-soit-A, where *T. brucei* was most abundant. The distribution of these species by village is as shown in Fig. 3. The highest number of trypanosome infections was documented in Kimotorok village (68/239), while least infections were found in Loibor-soit-A (26/239).

### Seasonal distribution of trypanosome infections

The overall prevalence of trypanosome infections in cattle was higher at the end of wet season (18.7%) than at the beginning of the wet season (16.5%) or in the dry season (16.0%). The seasonal variation was not statistically significant neither when the overall cattle population was considered nor when the cattle were split by village (Table 4). However, a significant seasonal variation of prevalence of individual trypanosome species was obtained when *T. brucei* and *T. theileri* were analysed (*P* = 0.025) (Table 5).

**Table 4:** Seasonal variation of trypanosome infections in cattle

Season	No. of cattle	Prevalence of infection, *n* (%)	*χ* ^2^	*P*-value
Early wet	200	33 (16.5)	1.0731	0.5848
End of wet	402	75 (18.7)		
Dry	400	64 (16.0)		
Total	1002	172 (17.2)

**Table 5:** Distribution of trypanosome infections across seasons

Species	Total no. of infections	No. of infections (%)			*χ* ^2^	*P*-value
		End of wet season	Dry season	Beginning of wet season		
*T. vivax*	100	11.9 (48)	10 (40)	6 (12)	5.25	0.072
*T. congolense*	18	2.49 (10)	1 (4)	2 (4)	2.58	0.275
*T. brucei*	50	5.47 (22)	3 (12)	8 (16)	7.36	0.025
*T. simiae*	47	6.22 (25)	4 (16)	3 (6)	3.81	0.149
*T. theileri*	24	3.98 (16)	1.5 (6)	1 (2)	7.36	0.025

### Prevalence of trypanosomes in cattle and tsetse flies

**Fig. 4:**
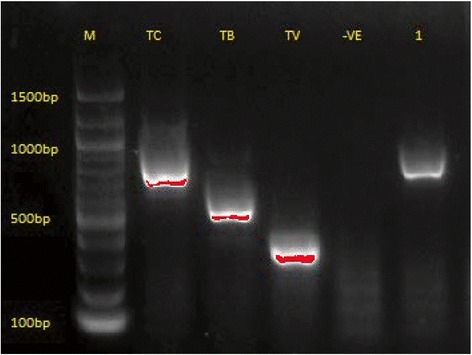
A gel of ITS1 single PCR. Lane M: DNA marker; Lanes TC, TB and TV: positive controls for *T. congolense*, *T. brucei* and *T. vivax*, respectively; Lane -VE: negative control; Lane 1: positive sample of *T. congolense* (700 bp)

Trypanosome infections were concurrently studied in cattle and tsetse flies in order to determine the variation of prevalence spatially. Table 6 shows the spatial variation in the two hosts and Fig. 4 shows the bands obtained using ITS 1 single PCR.

**Table 6:** Trypanosome prevalence in cattle and tsetse flies

Village	Cattle		Tsetse flies		*P*-value
	No. screened	No. of infections (%)	No. screened	No. of infections (%)	
Emboreet	100	13 (13.0)	72	10 (13.9)	0.8658
Kimotorok	100	21 (21.0)	9	0 (0)	0.1340
Loiborsiret	100	10 (10.0)	750	20 (2.7)	0.0016
Ortukai	102	10 (9.8)	55	0 (0)	0.0114
Total	402	54 (13.4)	886	30 (3.4)	< 0.0001

The overall prevalence of trypanosomes using a single step PCR for the ITS 1 gene was significantly higher in cattle (13.4%) than in tsetse flies (3.4%) (*P* < 0.0001) (Table 5). However, when prevalence in cattle and tsetse flies was examined in individual villages, a different trend was shown in different villages. For example, in Emboreet village the prevalence of trypanosomes was high in tsetse flies (13.9%) but slightly lower in cattle (13.0%) whereas in Kimotorok village, a 0% prevalence was shown in tsetse flies but 21.0% prevalence in cattle. *Trypanosoma vivax* was the most prevalent infection in tsetse flies (21/30; 70.0%) compared to that of *T. brucei* (6/30; 20.0%) and *T. congolense* (3/30; 10.0%). None of the tsetse flies collected during this period had mixed trypanosome infections, and none of the *T. brucei* positive DNA was positive for human-infective trypanosome species (*T. b. rhodesiense*).

## Discussion

This study established a 17.2% prevalence of trypanosome infections in cattle in the selected villages of the Maasai Steppe. The prevalence reflects trypanosome infections in cattle regardless of their clinical status at the time of sampling. The prevalence level further indicates the potential risk for pastoral cattle to suffer from trypanosomosis and spread the infections to other animals through tsetse fly bites. This prevalence is higher than the 5% reported by others [30] using parasitological (microscopy) methods to detect active disease in the same area. One of the reasons behind the difference in the prevalence is the sensitivity of PCR compared to microscopy in the detection of trypanosomes. Further, PCR detects both clinical and subclinical infections which include pathogenic and non-pathogenic trypanosomes. Only cattle infected with pathogenic trypanosomes would show clinical signs depending on the virulence of the infecting species involved. For example, cattle infected with *T. congolense* are more likely to show clinical signs than cattle infected with *T. theileri* [6, 31, 32]. The 17.2% prevalence of trypanosome infections reported in this study also reflects the high trypanosome transmission intensity in livestock-wildlife interface areas, as may be expected in the Maasai Steppe ecosystem, Several other studies have also reported a similar prevalence using PCR methods [21, 33, 34].

The present study compared the prevalence of trypanosome infections in different age groups of cattle. Higher prevalence of trypanosome infections was shown in cattle that were older than 2 years compared to younger ones, although the difference between age groups was not significant. Interestingly, a high strength of association was shown between age and infection prevalence (*χ* ^2^ = 32.9). This finding was expected since older cattle grazed in tsetse fly infested areas whereas younger animals grazed close to homesteads, hence their lower exposure to tsetse bites. Our study has shown a significant variation of prevalence of trypanosome infections between villages. The variation may be ascribed to differences in community practices for vector control between villages as well as livestock movement often towards protected areas with high abundance of wildlife. Seasonal livestock movement from one village to another in search of pasture and water is a common phenomenon in the Maasai Steppe, and such movements are expected to influence the prevalence of trypanosome infections. Furthermore, the movement of cattle herds increases their interaction with tsetse flies and wildlife through shared grazing fields and hence the potential for disease transmission between wild and domestic hosts [5]. Thus vector control is essential to cattle in these areas especially in villages like Loiborsoit-A where the prevalence of trypanosomes was high, and Kimotorok and Loiborsiret which are closest to Tarangire National park. The most common vector control method in the Maasai Steppe is the use of acaricide sprayers, which is not very cost-effective and is labour intensive. The ideal method would be to use dip tanks most of which are not functional due to structural damage and water shortage to refill the tanks. However, regular dipping of cattle before and after seasonal livestock movements to highly infested areas is essential [35, 36], what will complement already existing community-level vector control strategies and hence reduce trypanosome infections.

We have shown that the overall prevalence of trypanosome infections was higher at the end of wet season (18.7%) than during the dry and beginning of wet seasons. However, the seasonal variation was not significant. The non-significant overall variation of trypanosome infections could be due to chronicity of the parasites what can potentially lead to persistent trypanosome infections across seasons [37]. However, when individual trypanosome species were considered, significant seasonal variation was shown between *T. brucei* (whose prevalence was higher at the beginning of the wet season) and *T. theileri.* The variation of individual trypanosome species could also be due to biology and epidemiology of tsetse flies transmitting individual parasites. For example, *Glossina morsitans* is a good vector for *T. congolense* [38] while *G. pallidipes, G. morsitans* and *G. swynnertoni* are efficient vectors for *T. brucei* [5, 39]. *Trypanosoma theileri* is not dependent on the tsetse fly distribution but that of Tabanid flies [8]. In the Maasai Steppe, cattle come into close contact with the vectors either during normal grazing or movement due to seasonal changes in pasture availability. Another reason explaining the seasonal variation of the different trypanosome species is the potential mechanical transmission component of *T. congolense* and *T. vivax*, whose transmission is also dependant on tabanid and *Stomoxys* flies [40].

This study identified both pathogenic and non-pathogenic trypanosomes in cattle. The most prevalent trypanosome species was *T. vivax* which comprised 41.8% (100/239) of the overall infections. The abundance of *T. vivax* is consistent with its transmission pattern giving it more chances of infection in cattle. The high abundance of *T. vivax* and *T. brucei* could explain the persistent subclinical infections exhibited by cattle in the Maasai Steppe [41], compared to less prevalent infections by pathogenic *T. congolense* as suggested by our study.

We report that 33.1% (57/172) of trypanosome positive cattle harboured mixed infections of two up to four trypanosome species. Mixed infections in mammalian hosts increase utilisation for nutrients and immune components due to competition for space and resources by trypanosomes leading to clinical implications on the host. This finding suggests that cattle in such endemic areas like the Maasai Steppe go through rounds of infection and re-infection with different trypanosome species, hence the persistence and chronicity of infections. Further, local cattle breeds may have adapted to live with low doses of trypanosomes in their blood without showing any signs of infection. However, this does not exempt them from getting multiple trypanosome infections. Another reason behind the occurrence of mixed trypanosome infections is the ability of tsetse flies to carry more than one species of trypanosomes. This is attributed to the different developmental predilection sites of trypanosome species. For example, *T. congolense* develops in the midgut while *T. vivax* develops entirely in the proboscis. This means that one tsetse fly can transmit more than one trypanosome species [42]. In the present study, we found *T. vivax*/*T. simiae* combination as the most frequent mixed infection which appeared in 26.4% of the positive cattle in all five villages. *Trypanosoma simiae* is considered not infective for cattle and therefore not pathogenic in cattle but very pathogenic to pigs. The presence of this species in cattle is not a common finding but has been reported before [32, 43]. The impact of the presence of this trypanosome species on the epidemiology of the disease is unclear so far although it suggests that pigs could be at risk of infection if exposed to both *T. simiae*-infected cattle and tsetse flies. However, most Maasai people do not rare pigs and as such this risk may not be anticipated. Mixed infections by pathogenic strains would lead to the manifestation of clinical signs in an infected host while infection by non-pathogenic species would result in chronicity of sub-clinical infections [44]. The mixed trypanosome infections in cattle reported here are comparable to those found in tsetse flies [45], where *T. vivax*/*T. simiae*/*T. brucei* was most abundant.

As expected, the overall prevalence of trypanosome infections was lower in tsetse flies than in cattle mostly because one tsetse fly can transmit trypanosomes to more than one animal. This finding could also be due to the fact that majority of trapped tsetse flies are comprised of unfed flies [46, 47], which normally have a lower chance of carrying trypanosome infections. The epsilon traps used in this study are known to be selectively specific to savannah tsetse flies (*G. pallidipes* and *G. morsitans*) and hence may leave out other tsetse fly species responsible for transmitting some species of trypanosomes [48–50]. Nevertheless, the prevalence of trypanosome infections in tsetse flies found in this study was higher than the previously reported values [5, 51] in the same study area. Higher infection prevalence in cattle, however, is due to the fact that cattle undergo multiple tsetse fly bites and each biting tsetse fly can transmit infections to several hosts giving cattle a higher prevalence of infections. On the other hand, the prevalence of trypanosome infections in cattle and tsetse flies reported for individual villages shows an inconsistent relationship, suggesting that high infection rates in flies do not necessarily mean high prevalence of trypanosomes in cattle (Table 6). This finding is most likely associated with human activities such as livestock movements, vector and disease control practices, which in the Maasai Steppe can be unpredictable. These findings entail that infections vary with time of the year (season) and location. This study also established a zero prevalence of mixed trypanosome infections in tsetse flies as opposed to the results reported by others [5, 45]. This finding could have been momentary considering that the study was conducted within a period of one month (June). An intensive or temporal study of mixed infections in tsetse flies could yield more information. However, the absence of mixed infections in this study could also be attributed to self- regulation and inhibition that takes place in the vectors [52]. The variation of trypanosome infections between cattle and tsetse flies could guide the Maasai pastoral communities living in tsetse fly infested areas when to control the disease strategically. The current study also showed no human-infective trypanosomes in both cattle and tsetse flies. These results are comparable to other studies conducted in the same area by others [5, 41, 51] who found no human-infective trypanosomes in northern Tanzania. Other studies have reported the presence of human-infective trypanosome in areas close to our study area [11]. The absence of circulating human-infective trypanosomes in the study area does not, however, call for relaxed control and surveillance, since the presence of reservoir hosts signifies an existing risk of emergence of the human-infective trypanosomes. Thus our study emphasizes the importance of enhanced control efforts by integrating the use of tsetse fly traps, chemotherapeutic and chemoprophylactic drugs that control the prevalent trypanosome species (including *T. brucei*), on livestock to maximize vector control in areas where tsetse flies are most abundant.

### Limitations of the study

As much as ITS 1 primers produced baseline results for trypanosome infections, they amplified only 3 species of trypanosomes (*T. congolense, T. brucei and T. vivax*) and rarely showed mixed infections under our laboratory conditions. This could be one of the reasons for zero prevalence of mixed infections in tsetse flies. On the other hand, ITS nested primers yielded higher fragment resolution. However, the nested PCR also produced fragments which are not among the listed sizes for trypanosome species. Such fragments may represent novel trypanosome species, and further studies on these fragments are in progress to clarify the identity of these putative bands.

## Conclusions

In summary, this study has established the seasonal and spatial prevalence of trypanosome infections in cattle and tsetse flies in villages in Simanjiro and Monduli districts of the Maasai Steppe. Age, sex and season did not significantly influence the prevalence of trypanosome infections. However, the location of the villages in relation to proximity to wildlife showed positive influence on the prevalence of trypanosome infections, with a high number of cattle carrying mixed trypanosome infections. This study has evidenced the circulation of five trypanosome species and factors that influence their occurrence. Factors such as geographical distribution of all trypanosome species and tsetse flies can be used as a guide to improved control measures. The knowledge and awareness of trypanosome infection distribution will enhance concrete human based control measures by the Maasai communities. These findings can also help strengthen trypanosomosis control measures at regional and national levels through implementation of active surveillance systems.
